# Supplementary material for: General nonlinear Hall current in magnetic insulators beyond the quantum anomalous Hall effect
Source: Nat Commun. 2023 May 26;14:3053. doi: 10.1038/s41467-023-38734-9 (PMC10219989; doi:10.1038/s41467-023-38734-9)
Supplement: Supplementary file 1 — Supplementary Information [file 41467_2023_38734_MOESM1_ESM.pdf]

# Supplementary information: General nonlinear Hall current in magnetic insulators beyond the quantum anomalous Hall effect


Daniel Kaplan, Tobias Holder, and Binghai Yan*

*Department of Condensed Matter Physics, Weizmann Institute of Science, Rehovot 7610001, Israel*



(Dated: April 25, 2023)



In this supplementary information, we provide the derivation of the nonlinear Quantum anomalous Hall effect using the Kubo formalism, detail the model for twisted bilayer graphene that forms the basis for the numerical evaluation, discuss the gauge invariance of the correction, state for completeness the integer quantum Hall effect at linear order within our notation, and give further details on the properties of the nonlinear conductivity in a two-band model.


## DIAGRAMS AT 2ND ORDER

The diagrams which are the source of Eq. (2), derived through the diagrammatic formalism and solution of the Dyson equation are,

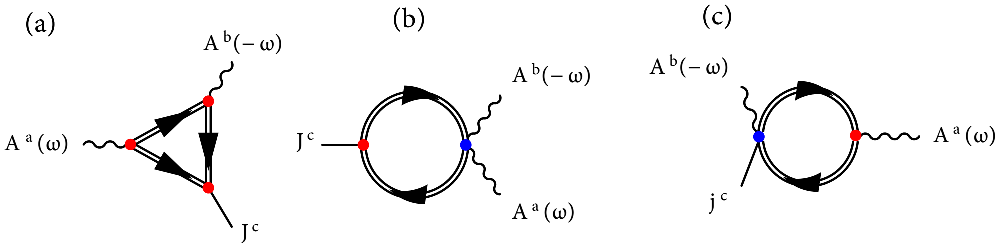


Supplementary Figure. S1. Contributing diagrams to the current $j^c$. There are three constituents, excluding symmetrization: (a) a triangle diagram, (b) 2nd order quadrupole vertex, (c) Renormalized current vertex. The red dots indicate a bare velocity vertex $v^a$. The blue indicate a 2nd order quadrupole vertex, $w^{ab}$. The definition of $v^a$ and $w^{ab}$ is given in the main text. Symmetrization of the form $(a,\omega) \leftrightarrow (b,-\omega)$ is necessary for the dc limit.

The diagrams in Fig. S2 contain a double-line Fermion propagator which represents the electron's retarded Green's function. The double line here stands for the dressing of this propagator with finite lifetimes. The proper prescription of lifetime inversion, elucidated in Ref. [1] requires distinguishing the projection of the self energy, depending on whether the Green's function connects intra-band or inter-band processes. For example, projecting the retarded Green's function on an intraband state, $\langle n\mathbf{k}|G_R(\omega)|n\mathbf{k}\rangle = \frac{1}{\omega+\frac{i\alpha}{\tau}}$. while inter-band transitions contain the bare lifetime $\tau$, $\langle n\mathbf{k}|G_R(\omega)|m\mathbf{k}\rangle = \frac{1}{\omega+(\varepsilon_n-\varepsilon_m)+\frac{i}{\tau}}$. By inspection of the semiclassical limit, we find that $\alpha = 2$. We define the terms originating from $(a)$ in Fig. S1 as $\mathcal{V}$, while the diagrams $(b)+(c)$ are grouped together as $\mathcal{W}$.

$$\mathcal{W}^{ab;c}_{nn} = \sum_m \frac{f_{nm} v^a_{nm} w^{bc}_{mn}}{(\omega+\frac{1}{\tau})(-\omega+\frac{1}{\tau})(\pm\omega+\varepsilon_{nm})+\frac{i}{\tau}} + \frac{1}{2}\sum_m \frac{f_{nm} w^{ab}_{nm} v^c_{mn}}{(\omega+\frac{1}{\tau})(-\omega+\frac{i}{\tau})\left(\varepsilon_{nm}+\frac{i\alpha}{\tau}\right)} \tag{S1}$$

$$\mathcal{V}^{ab;c}_{nn} = \sum_{m,l} f_{nm}\Bigg(\frac{v^a_{nm} v^b_{ml} v^c_{ln}}{(\omega+\frac{1}{\tau})(-\omega+\frac{1}{\tau})\left(\omega+\varepsilon_{nm}+\frac{i}{\tau}\right)\left(\varepsilon_{nl}+\delta_{nl}\frac{i\alpha}{\tau}+(1-\delta_{nl})\frac{i}{\tau}\right)} + \tag{S2}$$
$$\frac{v^c_{nl} v^a_{lm} v^b_{mn}}{(\omega+\frac{1}{\tau})(-\omega+\frac{1}{\tau})\left(-\omega-\varepsilon_{nm}+\frac{1}{\tau}\right)\left(\bar{\omega}-\varepsilon_{nl}+\delta_{nl}\frac{i\alpha}{\tau}+(1-\delta_{nl})\frac{i}{\tau}\right)}\Bigg).$$

Next, an expansion in powers of $\tau$ must be carried out to obtain the term which is of order $\tau^0$. Details of this are given in App. (B) of Ref. [1]. The reduction of the resulting terms is given in the next section.

## REDUCTION OF THE KUBO FORMULA

In this section we give the derivation of the electric-field induced nonlinear correction via a Kubo formula. The details of the diagrammatic approach are listed in Refs. [2]. We specifically the insertion of finite lifetimes according to the prescription of Ref. [1], and employ the notation therein. We expand to order $\tau^0$, which represents the dissipation-less correction to the linear conductivity. In the diagrammatic picture, 4 diagrams contribute. For simplicity, we consider here the case of $\sigma^{xx;y}$. An analogous expression can be derived for $\sigma^{yy;x}$. For compactness, we omit the Fermi occupation factor throughout. The total conductivity $\sigma^{xx;y}$ at order $\tau^0$ reads,

$$\sigma^{xx;y} = \mathcal{W}^{xx;y} + \mathcal{V}^{xx;y}. \tag{S3}$$

$\mathcal{W}^{xx;y}$ contains contributions from two-photon vertices, while $\mathcal{V}^{xx;y}$ are three-legged diagrams. Parsing $\mathcal{W}^{xx;y}$,

$$\mathcal{W}^{xx;y}_{\tau^0} = -2\left[\varepsilon^{-3}w^{xx}, v_y\right] - \left[\varepsilon^{-3}v^x, w^{xy}\right] = -2\left[\varepsilon^{-3}v_y, w^{xx}\right] - \left[\varepsilon^{-3}v^x, w^{xy}\right]. \tag{S4}$$

Recall that $w^{xx} = 2i\Delta^x\mathcal{A}^x - [\varepsilon\mathcal{A}^x, \mathcal{A}^x] + i\varepsilon S^{xx}$. Furthermore, $w^{xy} = i\Delta^x\mathcal{A}^y + i\Delta^y\mathcal{A}^x - \frac{1}{2}\left[\varepsilon\mathcal{A}^x, \mathcal{A}^y\right] - \frac{1}{2}\left[\varepsilon\mathcal{A}^y, \mathcal{A}^x\right] + i\varepsilon S^{xy}$. $v^{x,y} = i\varepsilon\mathcal{A}^{x,y}$ (since only off-diagonal components are involved).

$$\begin{aligned}\mathcal{W}^{xx;y}_{\tau^0} = &-2i\left[\varepsilon^{-2}\mathcal{A}^y, 2i\Delta^x\mathcal{A}^x - [\varepsilon\mathcal{A}^x, \mathcal{A}^x] + i\varepsilon S^{xx}\right] - \\ &i\left[\varepsilon^{-2}\mathcal{A}^x, i\Delta^x\mathcal{A}^y + i\Delta^y\mathcal{A}^x - (1/2)[\varepsilon\mathcal{A}^x, \mathcal{A}^y] - (1/2)[\varepsilon\mathcal{A}^y, \mathcal{A}^x] + i\varepsilon S^{xy}\right].\end{aligned} \tag{S5}$$

Terms may now be rearranged given the transposition properties of objects inside the commutator. For example, $\left[\varepsilon^{-2}\mathcal{A}^x, \Delta^x\mathcal{A}^y\right] = \left[\varepsilon^{-2}\mathcal{A}^y, \Delta^x\mathcal{A}^x\right]$. This stems from the fact that $\varepsilon^2_{nm} = \varepsilon^2_{mn}$, but $\Delta^x_{nm} = -\Delta^x_{mn}$. Thus,

$$\begin{aligned}\mathcal{W}^{xx;y}_{\tau^0} = &5\left[\varepsilon^{-2}\mathcal{A}^y, \Delta^x\mathcal{A}^x\right] + \left[\varepsilon^{-2}\mathcal{A}^x, \Delta^y\mathcal{A}^x\right] + 2i\left[\varepsilon^{-2}\mathcal{A}^y, [\varepsilon\mathcal{A}^x, \mathcal{A}^x]\right] - 2\left[\varepsilon^{-1}\mathcal{A}^y, S^{xx}\right] + \\ &\frac{i}{2}\left[\varepsilon^{-2}\mathcal{A}^x, [\varepsilon\mathcal{A}^x, \mathcal{A}^y] + [\varepsilon\mathcal{A}^y, \mathcal{A}^x]\right] - \left[\varepsilon^{-1}\mathcal{A}^x, S^{xy}\right].\end{aligned} \tag{S6}$$

Next we turn our to $\mathcal{V}^{xx;y}$. As the expressions contain denominators $\varepsilon_{nl}$ which depend on an intermediate index, we first isolate two cases of interest: $l = n$, $l = m$. The remainder are pieces for which $l \neq n, m$ and therefore are amenable to being written as proper commutators, as noted in the introduction. Since this section will involve explicit diagonal parts of the velocity operators $v^{x,y}_{nn}$ we restore the Fermi occupation factors. Firstly,

$$\mathcal{V}^{xx;y}_{l=n,\tau^0} = \frac{f_{nm}}{2\varepsilon^4_{nm}}v^x_{nm}v^x_{mn}v^y_{nn} + \frac{f_{nm}}{2\varepsilon^4_{nm}}v^y_{nn}v^x_{mn}v^x_{nm} \tag{S7}$$

Interchanging the summation on the second term ($n \leftrightarrow m$), gives,

$$\frac{f_{nm}}{2\varepsilon^4_{nm}}\left(v^x_{nm}v^x_{mn}(v^y_{nn} - v^y_{mm})\right) = \frac{1}{2}\left[\frac{v^x}{\varepsilon^4}\Delta^y, v^x\right] = \frac{1}{2}\left[\frac{\mathcal{A}^x}{\varepsilon^2}\Delta^y, \mathcal{A}^x\right]. \tag{S8}$$

Next is the case of $l = m$,

$$\mathcal{V}^{xx;y}_{l=m,\tau^0} = -7\frac{f_{nm}}{\varepsilon^4_{nm}}v^x_{nm}v^x_{mm}v^y_{mn} - 7\frac{f_{nm}}{\varepsilon^4_{nm}}v^y_{nm}v^x_{mm}v^x_{mn} = 7\left[\varepsilon^{-4}v^y\Delta^x, v^x\right] = 7\left[\varepsilon^{-2}\mathcal{A}^y\Delta^x, \mathcal{A}^x\right] = -7\left[\varepsilon^{-2}\mathcal{A}^y, \Delta^x\mathcal{A}^x\right]. \tag{S9}$$

The remaining terms, such that $l \neq n, m$ are,

$$\mathcal{V}^{xx;y,(1)}_{l\neq n,m,\tau^0} = -4\frac{f_{nm}}{\varepsilon_{nm}\varepsilon^3_{nl}}v^x_{nm}v^x_{ml}v^y_{ln} - 2\frac{f_{nm}}{\varepsilon^2_{nm}\varepsilon^2_{nl}}v^x_{nm}v^x_{ml}v^y_{ln} - \frac{f_{nm}}{\varepsilon^3_{nm}\varepsilon_{nl}}v^x_{nm}v^x_{ml}v^y_{ln}, \tag{S10}$$

while $\mathcal{V}^{xx;y,(2)}_{l\neq n,m}$ is the complex conjugate of $\mathcal{V}^{xx;y,(1)}_{l\neq n,m}$. Let us treat each term separately. After substituting $v^{x,y}$, and adding the complex conjugate the first term yields $-4\frac{f_{nm}}{\varepsilon_{nm}\varepsilon^3_{nl}}v^x_{nm}v^x_{ml}v^y_{ln} = -4i\left[\mathcal{A}^x, \left[\varepsilon\mathcal{A}^x, \varepsilon^{-2}\mathcal{A}^y\right]\right]$. We break this term up into two pieces of equal prefactor, 2, and the first piece is replaced using a Jacobi identity. That is, $-2i\left[\mathcal{A}^x, \left[\varepsilon\mathcal{A}^x, \varepsilon^{-2}\mathcal{A}^y\right]\right] = 2i\left[\varepsilon^{-2}\mathcal{A}^y, \left[\mathcal{A}^x, \varepsilon\mathcal{A}^x\right]\right] + 2i\left[\varepsilon\mathcal{A}^x, \left[\varepsilon^{-2}\mathcal{A}^y, \mathcal{A}^x\right]\right]$. The other piece is written down differently. Using the fact that $\varepsilon_{ml} = \varepsilon_{mn} + \varepsilon_{nl}$, $-2i\left[\mathcal{A}^x, \left[\varepsilon\mathcal{A}^x, \varepsilon^{-2}\mathcal{A}^y\right]\right] = -2i\left[\mathcal{A}^x, \varepsilon\left[\mathcal{A}^x, \varepsilon^{-2}\mathcal{A}^y\right]\right] + 2i\left[\mathcal{A}^x, \left[\mathcal{A}^x, \varepsilon^{-1}\mathcal{A}^y\right]\right]$. Adding this up once more gives,

$$-4i\left[\mathcal{A}^x, \left[\varepsilon\mathcal{A}^x, \varepsilon^{-2}\mathcal{A}^y\right]\right] = 2i\left[\varepsilon^{-2}\mathcal{A}^y, [\mathcal{A}^x, \varepsilon\mathcal{A}^x]\right] + 2i\left[\mathcal{A}^x, \left[\mathcal{A}^x, \varepsilon^{-1}\mathcal{A}^y\right]\right]. \tag{S11}$$

The next term in Eq. (S10) is decomposed analogously.

$$-2\frac{f_{nm}}{\varepsilon_{nm}^2\varepsilon_{ln}^2}v_{nm}^x v_{ml}^x v_{ln}^y = 2i\left[\frac{\mathcal{A}^x}{\varepsilon},\left[\varepsilon\mathcal{A}^x,\varepsilon^{-1}\mathcal{A}^y\right]\right] = -2i\left[\mathcal{A}^x,\left[\mathcal{A}^x,\varepsilon^{-1}\mathcal{A}^y\right]\right] - 2i\left[\varepsilon^{-1}\mathcal{A}^x,\left[\mathcal{A}^x,\mathcal{A}^y\right]\right] \tag{S12}$$

The last term in Eq. (S10) is given by,

$$-\frac{f_{nm}}{\varepsilon_{nm}^3\varepsilon_{nl}}v_{nm}^x v_{ml}^x v_{ln}^y = -i\left[\varepsilon^{-2}\mathcal{A}^x,\left[\varepsilon\mathcal{A}^x,\mathcal{A}^y\right]\right]. \tag{S13}$$

We are now ready to assemble all the pieces we have. We combine,

$$\begin{aligned}
&\mathcal{V}^{xx;y} + \mathcal{W}^{xx;y} = \\
&5\left[\varepsilon^{-2}\mathcal{A}^y,\Delta^x\mathcal{A}^x\right] + \left[\varepsilon^{-2}\mathcal{A}^x,\Delta^y\mathcal{A}^x\right] + 2i\left[\varepsilon^{-2}\mathcal{A}^y,\left[\varepsilon\mathcal{A}^x,\mathcal{A}^x\right]\right] - 2\left[\varepsilon^{-1}\mathcal{A}^y,S^{xx}\right] + \\
&\frac{i}{2}\left[\varepsilon^{-2}\mathcal{A}^x,\left[\varepsilon\mathcal{A}^x,\mathcal{A}^y\right] + \left[\varepsilon\mathcal{A}^y,\mathcal{A}^x\right]\right] - \left[\varepsilon^{-1}\mathcal{A}^x,S^{xy}\right] + \frac{1}{2}\left[\frac{\mathcal{A}^x}{\varepsilon^2}\Delta^y,\mathcal{A}^x\right] - 7\left[\varepsilon^{-2}\mathcal{A}^y,\Delta^x\mathcal{A}^x\right] + 2i\left[\varepsilon^{-2}\mathcal{A}^y,\left[\mathcal{A}^x,\varepsilon\mathcal{A}^x\right]\right] \\
&+ 2i\left[\mathcal{A}^x,\left[\mathcal{A}^x,\varepsilon^{-1}\mathcal{A}^y\right]\right] - 2i\left[\mathcal{A}^x,\left[\mathcal{A}^x,\varepsilon^{-1}\mathcal{A}^y\right]\right] - 2i\left[\varepsilon^{-1}\mathcal{A}^x,\left[\mathcal{A}^x,\mathcal{A}^y\right]\right] - i\left[\varepsilon^{-2}\mathcal{A}^x,\left[\varepsilon\mathcal{A}^x,\mathcal{A}^y\right]\right].
\end{aligned} \tag{S14}$$

We obtain,

$$\begin{aligned}
&\mathcal{V}^{xx;y} + \mathcal{W}^{xx;y} = \\
&-2\left[\varepsilon^{-2}\mathcal{A}^y,\Delta^x\mathcal{A}^x\right] + \frac{1}{2}\left[\varepsilon^{-2}\mathcal{A}^x,\Delta^y\mathcal{A}^x\right] - 2\left[\varepsilon^{-1}\mathcal{A}^y,S^{xx}\right] + \frac{i}{2}\left[\varepsilon^{-2}\mathcal{A}^x,-\left[\varepsilon\mathcal{A}^x,\mathcal{A}^y\right] + \left[\varepsilon\mathcal{A}^y,\mathcal{A}^x\right]\right] + \\
&-\left[\varepsilon^{-1}\mathcal{A}^x,S^{xy}\right] - 2i\left[\varepsilon^{-1}\mathcal{A}^x,\left[\mathcal{A}^x,\mathcal{A}^y\right]\right].
\end{aligned} \tag{S15}$$

This term contains several total derivatives, which are Fermi surface terms, and give rise to the recently proposed gravitational anomaly and intrinsic non-dissipative Hall effects [3, 4]. We remove them by observing that,

$$\partial_y\left[\varepsilon^{-1}\mathcal{A}^x,\mathcal{A}^x\right] = 2\left[\varepsilon^{-1}\mathcal{A}^x,S^{xy}\right] + \left[\varepsilon^{-2}\mathcal{A}^x,\Delta^y\mathcal{A}^x\right] + \left[\varepsilon^{-1}\mathcal{A}^x,i[\mathcal{A}^y,\mathcal{A}^x]\right] \tag{S16}$$

$$\partial_x\left[\varepsilon^{-1}\mathcal{A}^y,\mathcal{A}^x\right] = \left[\varepsilon^{-1}\mathcal{A}^x,S^{xy}\right] + \left[\varepsilon^{-1}\mathcal{A}^y,S^{xx}\right] - \left[\varepsilon^{-2}\mathcal{A}^y\Delta^x,\mathcal{A}^x\right] + \left[\varepsilon^{-1}\mathcal{A}^x,(i/2)[\mathcal{A}^x,\mathcal{A}^y]\right]. \tag{S17}$$

By combining these identities with the symmetrization condition one finds,

$$\begin{aligned}
&\sigma^{xx;y} = 2\partial_y\left[\varepsilon^{-1}\mathcal{A}^x,\mathcal{A}^x\right] - \partial_x\left[\varepsilon^{-1}\mathcal{A}^y,\mathcal{A}^x\right] + \\
&\frac{1}{2}\left[\varepsilon^{-2}\mathcal{A}^y,\Delta^x\mathcal{A}^x\right] - \frac{1}{2}\left[\varepsilon^{-2}\mathcal{A}^x,\Delta^y\mathcal{A}^x\right] + \left[\varepsilon^{-1}\mathcal{A}^x,S^{xy}\right] - \left[\varepsilon^{-1}\mathcal{A}^y,S^{xx}\right] + \left[\varepsilon^{-1}\mathcal{A}^x,(i/2)[\mathcal{A}^x,\mathcal{A}^y]\right].
\end{aligned} \tag{S18}$$

In the above, we employed the identity that $\tilde{\Omega}^{xy,1} - \tilde{\Omega}^{yx,1} = \varepsilon\Omega^{ab}$, otherwise proven in [1]. We note that this form is fully compatible with the consistent separation of nonlinear response conductivity into Hall components carried out in Ref. [5]. We also note that the positional shift $S^{xy}$ described here is related (while more general) to the quantum metric dipole, also recently shown to present an in-gap Hall conductivity [6].

## PARSING OF CONDUCTIVITIES IN COMPACT FORM

Eqs. (4)-(6) in the main text are given in compact notation involving commutators and operators with explicit band projections. In this section, we illustrate how these terms should be understood in terms of Bloch states $|n\mathbf{k}\rangle = |n\rangle$, scalar quantities such as $\Delta^x$ and band energy difference $\varepsilon_n - \varepsilon_m$. We first note that the commutator, expressed in the Bloch for any two operators $A, B$ should be read as,

$$\langle n\mathbf{k}|[A,B]|m\mathbf{k}\rangle = [A,B]_{nm} = \sum_{l\neq n,m}\left(A_{nl}B_{lm} - B_{nl}A_{lm}\right). \tag{S19}$$

The objects appearing in the main text are constructed such that the projection manifold of all intermediate states $l$ is separated from $n, m$. For simplicity (since all symmetries are broken), states are assumed to be non-degenerate. If the commutator is weighted by a Fermi-Dirac factor of a given band $n$, $f_n$, the commutator can take a simpler form,

$$\sum_n f_n[A,B]_{nn} = \sum_{n,l\neq n} f_n\left(A_{nl}B_{ln} - B_{nl}A_{ln}\right) = \sum_{n\neq l}(f_n - f_l)A_{nl}B_{ln}. \tag{S20}$$

Here, we used the summation over all states $n$ to exchange the summation dummy variable $l \leftrightarrow n$. All scalar differences, such as $f_n - f_l = f_{nl}$ for compactness. This applies to other scalar objects, as $\varepsilon_n - \varepsilon_m = \varepsilon_{nm}$. $\Delta^x_{nm} = v^x_{nn} - v^y_{mm}$. $\varepsilon_n$ is the energy of Bloch state $n$ and $v^x_{nn}$ is the velocity in the $x$ direction of band $n$. All scalars that multiply Hermitian operators enter only as Hadamarad products. In other words, the combination $(A\Delta^x)_{nm} = 0\delta_{nm} + (1-\delta_{nm})A_{nm}\Delta^x_{nm}$. An object appearing in the denominator must be as a scalar. For example, $(\varepsilon^{-1}A)_{nm} = 0\delta_{nm} + \frac{(1-\delta_{nm})A_{nm}}{\varepsilon_n - \varepsilon_m} = 0\delta_{nm} + \frac{(1-\delta_{nm})A_{nm}}{\varepsilon_{nm}}$. Note that this expression is always regular, as it is non-zero if and only if $n \neq m$. Assuming there are no systematic degeneracies, $\varepsilon_n \neq \varepsilon_m$. Taking $I_1$ as an example,

$$I_1 = \sum_{n \in \text{occ.}} \left[\varepsilon^{-2}\mathcal{A}^x, \Delta^y \mathcal{A}^x\right]_{nn} = \sum_{n,m \neq n} \frac{\mathcal{A}^x_{nm}}{(\varepsilon_n - \varepsilon_m)^2}\Delta^y_{mn}\mathcal{A}^x_{mn} - \Delta^y_{nm}\mathcal{A}^x_{nm}\frac{\mathcal{A}^x_{mn}}{(\varepsilon_m - \varepsilon_n)^2} =$$
$$\frac{\mathcal{A}^x_{nm}}{(\varepsilon_n - \varepsilon_m)^2}(v^y_{mm} - v^y_{nn})\mathcal{A}^x_{mn} - (v^y_{nn} - v^y_{mm})\mathcal{A}^x_{nm}\frac{\mathcal{A}^x_{mn}}{(\varepsilon_m - \varepsilon_n)^2}. \tag{S21}$$

In a similar fashion, this same form of expansion is carried over to other terms in $I_2$ and $I_3$ respectively.

## MODEL FOR TWISTED BILAYER GRAPHENE

The numerical calculation presented in Fig. 2 for twisted bilayer graphene at $\nu = \frac{3}{4}$ filling is carried out on strained version of the continuum model [7] (B-M model). The presence of the strain-induced gauge field, as well as the modification of the tunneling elements in the model induce additional couplings between states which then produce a non-zero nonlinear conductivity. The basic model construction consists of two graphene sheets, with Hamiltonians $H_t, H_b$, standing for the (t)op and (b)ottom layers respectively. The twisting of one layer with respect to another is represented by the operator $\mathbf{R}_{t,b}$, which rotates the underlying Pauli matrix basis of either $H_t$ or $H_b$ by an angle $+\frac{\theta}{2}, -\frac{\theta}{2}$, respectively. the Hamiltonian has the form,

$$H_{t,b} = \hbar v_f \sum_q c^\dagger_{q,t/b}\mathbf{R}\left(\mathbf{q}\cdot\boldsymbol{\sigma}\right)c_{q,t/b}. \tag{S22}$$

Here $c_{q,t/b}$ are standard creation/annihilation operators in every layer. Following the model introduced in App. A of Ref. [8], we introduce coupling between the layers, via the Hamiltonian,

$$H_{t\to b} = \sum_{q,q'} c^\dagger_t(q)(T_{1,q,q'} + T_{2,q,q'} + T_{3,q,q'})c_b(q'). \tag{S23}$$

The matrices $T_1, T_2, T_3$ are the tunneling elements precisely as they are defined in the B-M picture. At this level, the model retains inversion from the continuum description of monolayer graphene. It also has $C_{3z}$ symmetry. To break these two symmetries, we consider first a sub-lattice potential which we imagine creates spontaneous layer polarization, due to alignment with hBN.

$$H_{\text{hBN}} = \sum_q c^\dagger_{q,b}\left(\Delta\sigma_z\right)c_{q,b}. \tag{S24}$$

Here $\Delta = 17$meV. Strain is introduced via the modification of the tunneling elements $T_{1\ldots3}$, as explained in App. B of Ref. [8]. Additionally a pseudo gauge field originating from strain, which is due to the shift of the original $K$ points of monolayer graphene,

$$\tilde{K} = (1 - \epsilon^T)K \mp \tilde{A}, \tag{S25}$$

where $\epsilon = \epsilon\left(\begin{smallmatrix}-1 & 0\\ 0 & 1\end{smallmatrix}\right)$ is the uniaxial strain tensor and $\epsilon$ is its magnitude. $K$ are the original Dirac points of graphene, and $\tilde{A} = -\frac{\beta\epsilon}{d}(1+\nu, 0)$ is the pseudo gauge field that stems from uniaxial strain [9]. The constants $\beta = 1.57$ and $\nu = 0.165$ are known [10]. The strain breaks $C_{3z}$ and essentially all other crystalline symmetries permitting non-zero nonlinear response. The 3/4 filling case is obtained by taking only one Dirac point of the original graphene thus explicitly breaking time-reversal symmetry.

## GAUGE INVARIANCE OF THE DERIVED CORRECTION

The introduction of derivatives of the wavefunction in the expressions for $I_1 - I_3$ Eqs. (4)-(6) in the main text requires delicate handling of gauge transformations. The Bloch manifold of cell-periodic states is characterized by an invariance to the $U(1)^N$ transformation,

$$|n\mathbf{k}\rangle \to e^{-i\theta_n(k)} |n\mathbf{k}\rangle . \tag{S26}$$

For notational ease, we suppress below the label $\mathbf{k}$, and refer to $|n\rangle$, as $|n\rangle = |n\mathbf{k}\rangle$. The Bloch periodic part of the Hamiltonian commutes with this gauge transformation since $[H(\mathbf{k}), f(k)] = 0$. The observables of optical response are modified due to the gauge covariance of the states. The velocity operator $v^\alpha \to Uv^\alpha$, where $U_{nm} = e^{i\theta_{nm}}$, $\theta_{nm} = \theta_n - \theta_m$. Clearly, only diagonal components, such as those comprising $\Delta^\alpha_{nm} = v^\alpha_{nn} - v^\alpha_{mm}$, are automatically gauge invariant. Products such as $(v^\alpha)^\dagger v^\beta \to v^\alpha U^\dagger U v^\beta = v^\alpha v^\beta$ are gauge invariant. In this respect, the equations of optical response in the velocity gauge are manifestly gauge invariant, as they combine products of gauge covariant operators derived from the Hamiltonian. The issue of local $U(1)$ gauge invariance in the context of electromagnetism and optical response has recently been addressed, with gauge invariance formally proven [11–14]. Our primary focus, therefore, is to show that Eqs. (4)-(6)which involve *derivatives* of the Bloch periodic part of the electronic wavefunctions are also gauge invariant. The principle derivatives defining 2nd order optical response are [1]:

$$\mathcal{A}^\alpha_{nm} = \langle n| i\partial_\alpha |m\rangle \to \partial_\alpha\theta_n\delta_{nm} + e^{i\theta_{nm}}\langle n| i\partial_\alpha |m\rangle = e^{i\theta_{nm}}\mathcal{A}^\alpha_{nm} + \partial_\alpha\theta_n\delta_{nm} \tag{S27}$$

$$\lambda^{\alpha\beta}_{nm} = \frac{1}{2}\langle n| i\partial_\alpha\partial_\beta |m\rangle + (\text{c.c.}, n \leftrightarrow m) \to e^{i\theta_{nm}}\lambda^{\alpha\beta}_{nm} + \frac{ie^{i\theta_{nm}}}{2}\left(\mathcal{A}^\alpha_{nm}\partial_\beta\theta_{nm} + \mathcal{A}^\beta_{nm}\partial_\alpha\theta_{nm}\right), \; n \neq m. \tag{S28}$$

We define objects which are gauge *covariant* as those which transform as $A_{nm} \to A_{nm}e^{i\theta_{nm}}$. Consequently, combinations of the form $A_{nm}A_{mn}$ are manifestly gauge *invariant* since they transform like the velocity operator, as shown above, or $e^{i\theta_{nm}}A_{nm}e^{i\theta_{mn}}A_{mn} = A_{nm}A_{mn}$. Generally, for *any* two covariant objects $A, B$, the commutator of the two satisfies,

$$[A, B]_{nm} \to e^{i\theta_{nm}}[A, B]_{nm}, \tag{S29}$$

rendering its diagonal part gauge invariant. This follows from the definition introduced in the main text, $[A, B]_{nm} = \sum_{l\neq n,m} A_{nl}B_{lm} - (A \leftrightarrow B)$. We now prove that the quantity $S^{\alpha\beta}_{nm}, n \neq m$ is gauge covariant, which appears in $I_2$, of Eq. (5). We note that $S^{\alpha\beta}$ consists of two portions: $\lambda^{\alpha\beta}_{nm}$ and the Hadamard product, $\frac{i}{2}\mathcal{A}^\alpha_{nm}\delta^\beta_{nm} + (\alpha \leftrightarrow \beta)$. Tackling the latter first,

$$\frac{i}{2}\mathcal{A}^\alpha_{nm}\delta^\beta_{nm} \to \frac{i}{2}e^{i\theta_{nm}}\mathcal{A}^\alpha_{nm}\partial_\beta\theta_{nm}. \tag{S30}$$

To uncover the transformation properties of $\lambda^{\alpha\beta}_{nm}$ for $n \neq m$, we first observe that under $|m\rangle \to e^{i\theta_m}|m\rangle$, $\partial_\alpha\partial_\beta|m\rangle \to e^{i\theta_m}(i\partial_\alpha\theta_m|\partial_\beta m\rangle + i\partial_\beta\theta_m|\partial_\alpha m\rangle + |\partial_\alpha\partial_\beta m\rangle)$. We explicitly remove the term $e^{i\theta_m}\partial_\alpha\partial_\beta\theta_m|m\rangle$ since we assume that $n \neq m$ and this contribution must vanish when projected back onto the Bloch states. Multiplying on the left with $i\langle n|$, we find that,

$$\lambda^{\alpha\beta}_{nm} \to \frac{e^{i\theta_{nm}}}{2}\lambda^{\alpha\beta}_{nm} + \frac{i}{2}e^{i\theta_{nm}}\mathcal{A}^\alpha\partial_\beta\theta_{mn} + (\alpha \leftrightarrow \beta). \tag{S31}$$

It follows that the sum of the objects,

$$\frac{1}{2}\lambda^{\alpha\beta}_{nm} + \frac{i}{2}\mathcal{A}^\alpha_{nm}\delta^\beta_{nm} + (a \leftrightarrow b) \to e^{i\theta_{nm}}(\frac{1}{2}\lambda^{\alpha\beta}_{nm} + \frac{i}{2}\mathcal{A}^\alpha_{nm}\delta^\beta_{nm}) + (a \leftrightarrow b), \tag{S32}$$

With the $\theta$ dependent part explicitly cancelling as it appears with opposite indices $\theta_{nm}$ vs $\theta_{mn}$. Lastly, the triple commutator introduced in Eq. (6) transforms using the rules outlined above for commutators. The product reads,

$$[\mathcal{A}^x, i[\mathcal{A}^x, \mathcal{A}^y]]_{nn} = i\mathcal{A}^x_{nm}[\mathcal{A}^x, \mathcal{A}^y]_{mn} - i[\mathcal{A}^x, \mathcal{A}^y]_{nm}\mathcal{A}^x_{mn} \to e^{i\theta_{nm}}\mathcal{A}^x_{nm}e^{i\theta_{mn}}[\mathcal{A}^x, \mathcal{A}^y]_{mn} - (\text{c.c.}) = [\mathcal{A}^x, i[\mathcal{A}^x, \mathcal{A}^y]]_{nn}. \tag{S33}$$

Here, we used the fact that commutator as defined does not sum over any diagonal contributions and off-diagonal parts of the Berry connection transform according to Eq. (S27).

## QUANTIZATION OF THE LINEAR KUBO FORMULA FOR THE IQHE

The linear response contribution at zero frequency reads [15],

$$\sigma^{xy} = \frac{ie^2}{L_x L_y \hbar} \sum_{n,m} f_{nm} \frac{v^x_{nm} v^y_{mn}}{\varepsilon^2_{nm}}. \tag{S34}$$

Here the sum $n, m$ runs over all bands (occupied and empty) including the degenerate manifold of each occupied Landau level. Based on the identities defined in the main text for the velocity matrix elements, we have,

$$v^x_{nm} v^y_{mn} = \frac{i\hbar\omega_c}{2M} \left(\sqrt{m+1}\delta_{n,m+1} - \sqrt{m}\delta_{n,m-1}\right)\left(\sqrt{m+1}\delta_{n,m+1} + \sqrt{m}\delta_{n,m-1}\right). \tag{S35}$$

The energy difference (measured in frequency units) is $\varepsilon_{nm} = \omega_c(n-m)$. The only surviving terms above are products of equal delta functions. Thus,

$$\frac{v^x_{nm} v^y_{mn}}{\varepsilon^2_{nm}} = \frac{i\hbar\omega_c}{2M} \frac{((m+1)\delta_{n,m+1} - m\delta_{n,m-1})}{\omega_c^2 (n-m)^2}. \tag{S36}$$

Assume now that there are $\nu$ occupied bands, which are each $N = \frac{eBL_xL_y}{h}$-fold degenerate. The linear conductivity Eq. (S34) has a global Fermi occupation factor difference $f_{nm} = f_n - f_m$. In the bulk of the quantum Hall fluid, a fully flat Landau band is either completely occupied or completely empty. Therefore, $f_{nm}$ is non-zero only in the cases $n = \nu, m = \nu+1, f_{nm} = 1$, and $n = \nu+1, m = \nu, f_{nm} = -1$. By further accounting for the degeneracy, we have,

$$\begin{aligned} \sigma^{xy} &= \frac{ie^2}{L_x L_y \hbar} \sum_{n,m} f_{nm} \frac{v^x_{nm} v^y_{mn}}{\varepsilon^2_{nm}} = \\ &\frac{eBL_xL_y}{h} \times \left[\frac{ie^2}{L_xL_y\hbar}\frac{i\hbar\omega_c}{2M}\left(\frac{-(\nu+1)}{\omega_c^2} - \frac{(\nu+1)}{\omega_c^2}\right)\right] = \frac{e^2}{h}(\nu+1). \end{aligned} \tag{S37}$$

$\sigma^{xy}$ is therefore quantized by the number of filled Landau levels.

## TWO BAND MODELS

The simplest approximation that can be made consists of a two band topological model with non-vanishing Berry curvature. We show here that a fundamental condition for the emergence of our derived correction is the presence of nonlinear terms. A minimal Hamiltonian where we find non-zero corrections reads,

$$H = (M + 2 - \cos(k_x) - \cos(k_y))\sigma_z + (\sin(k_x) + \alpha L_x)\,\sigma_x + (\sin(k_y) + \beta L_y)\,\sigma_y \tag{S38}$$

$$L_x = \sin(k_x)\sin(k_y),\ L_y = \sin(k_x)\cos(k_y), \tag{S39}$$

which is a model for a single Dirac cone with nonlinear terms represented by $L_x, L_y$. We stress that these non-linearities are manifested through higher-order momentum coupling, such as $\sin(k_x)\sin(k_y)$. Here, $\sigma$ are Pauli matrices representing an orbital basis. When $\alpha = \beta = 0$, the model has inversion symmetry, which is defined by $P = \sigma_z(k \to -k)$. This remains true even when $\alpha = 0, \beta \neq 0$, since $L_y$ is odd under $(k \to -k)$. The model is topological for all $-2 < M < 0$ with Chern number $C_N = -1$, and has $C_N = 0$ otherwise. Time reversal symmetry is explicitly broken. The nonlinearities $L_x, L_y$ are necessary not merely for inversion symmetry breaking. Since we require all point-group symmetries to be broken, perpendicular mirror symmetries cannot exist in the model. The mirrors in this model are broken by $L_x, L_y$. In addition, $L_x, L_y$ facilitate higher-order derivatives of the Hamiltonian, i.e., $w^{ab} = \partial_a\partial_b H_0$, thereby inducing a correction to the current operator (diagrams (b)+(c) in Supplementary Fig. S1), in accordance with Eq. (2). In the absence of non-linearities, sum rules [16], notably that the fact that $\partial_a v^b_{nm} = i[r^a, v^b]_{nm} + iv^b_{nm}\delta^a_{nm} + ir^a_{nm}\Delta^b_{nm}$, enforcing cancellations between terms giving a zero correction. In Supplementary Fig. S2(b), we show the magnitude of the correction as a function of of the mass parameter $M$ of the model. The response diverges as $M \to 0$ due to the presence of $\varepsilon^{-n}_{nm}$, $n > 1$ in all terms. Unlike the Berry curvature, the flux of this expression, i.e. the integral $\sim \int \mathrm{d}k\varepsilon^{-n}k$ does not equal a constant but decays as $k^{-3}$ in the leading order. This makes the correction fundamentally different from the Berry curvature, as it is highly singular in the limit $k \to 0$ for a vanishing mass. The corrections are sensitive to the topology of the system, as shown in Supplementary Fig. S2(b). In the non-topological case ($M > 0$), with

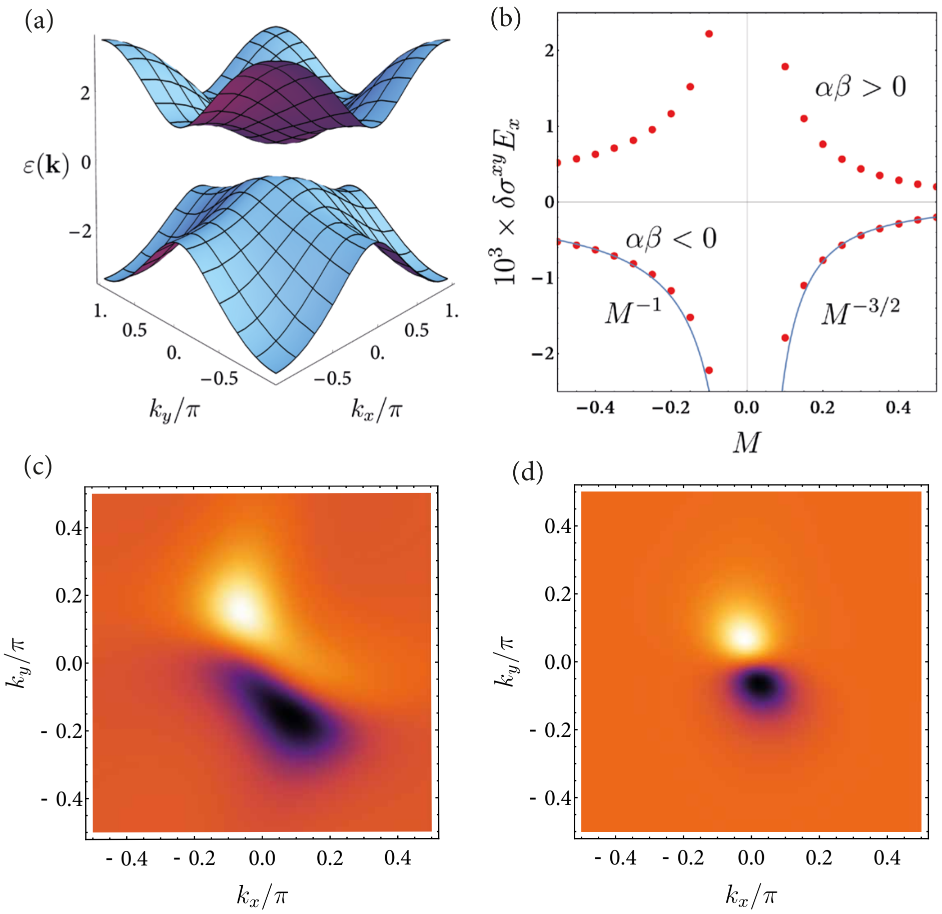


Supplementary Figure. S2. Nonlinear correction to the anomalous Hall conductivity of a gapped Dirac cone. (a) A two band model (Eq. (S39)) in the topological phase, when $M = -0.5$. (b) Magnitude of the correction $\delta\sigma^{xy}$ to the Hall conductivity, for $E_x = 1$. The sign of the correction depends on the sign of the product $\alpha\beta$. In the topological phase ($M < 0$), the correction decays like $M^{-1}$, while in the trivial phase ($M > 0$) it decays as $M^{-3/2}$. (c-d) Momentum space distribution of $I_1 - I_3$ (Eqs. (4)-(6)) for the topological phase (c) and the trivial phase (d)

vanishing total Berry phase, the corrections decay roughly as $M^{-3/2}$ while for $M < 0$, the decrease is of the form $M^{-1}$, indicating a slower suppression. The momentum space distribution is also different for the two cases. In Supplementary Fig. S2(c-d) we plot the momentum space density of the terms $I_1 - I_2$ (Eqs. (4)-(6)). In the trivial case ($M > 0$, Supplementary Fig. S2(d)), the divergence around the $\Gamma$ point of the model is clearly apparent, while when $M < 0$ the density is diffused across a broader region in momentum space, with the peak way from the $\Gamma$ point. As stated above, in the 2-band limit, $I_3$ (Eq. (6)) cannot contribute, and the non-linearity is most significantly encountered for the velocity shift $I_1$ (Eq. (4)).

* binghai.yan@weizmann.ac.il


[S1] D. Kaplan, T. Holder, and B. Yan, Unifying semiclassics and quantum perturbation theory at nonlinear order (2022a).

[S2] T. Holder, D. Kaplan, and B. Yan, Consequences of time-reversal-symmetry breaking in the light-matter interaction: Berry curvature, quantum metric, and diabatic motion, Phys. Rev. Research **2**, 033100 (2020), arXiv:1911.05667 [cond-mat.mes-hall].

[S3] T. Holder, D. Kaplan, R. Ilan, and B. Yan, Mixed axial-gravitational anomaly from emergent curved spacetime in nonlinear charge transport, arXiv (2021), 2111.07780.

[S4] Y. Gao, S. A. Yang, and Q. Niu, Field Induced Positional Shift of Bloch Electrons and Its Dynamical Implications, Phys. Rev. Lett. **112**, 166601 (2014), arXiv:1402.2538 [cond-mat.mes-hall].

[S5] S. S. Tsirkin and I. Souza, On the separation of Hall and Ohmic nonlinear responses, SciPost Phys. Core **5**, 39 (2022).

[S6] S. Lahiri, K. Das, D. Culcer, and A. Agarwal, Intrinsic nonlinear conductivity induced by the quantum metric dipole, , arXiv:2207.02178 (2022), arXiv:2207.02178 [cond-mat.mes-hall].
[S7] R. Bistritzer and A. H. MacDonald, Moiré bands in twisted double-layer graphene, PNAS **108**, 12233 (2011), arXiv:1009.4203 [cond-mat.mes-hall].
[S8] D. Kaplan, T. Holder, and B. Yan, Twisted photovoltaics at terahertz frequencies from momentum shift current, Phys. Rev. Research **4**, 013209 (2022b), arXiv:2101.07539 [cond-mat.mes-hall].
[S9] V. M. Pereira, A. H. Castro Neto, and N. M. R. Peres, Tight-binding approach to uniaxial strain in graphene, Phys. Rev. B **80**, 045401 (2009).
[S10] W.-Y. He, D. Goldhaber-Gordon, and K. T. Law, Giant orbital magnetoelectric effect and current-induced magnetization switching in twisted bilayer graphene, Nat. Commun. **11**, 1650 (2020).
[S11] G. B. Ventura, D. J. Passos, J. M. B. Lopes dos Santos, J. M. Viana Parente Lopes, and N. M. R. Peres, Gauge covariances and nonlinear optical responses, Phys. Rev. B **96**, 035431 (2017), arXiv:1703.07796 [cond-mat.other].
[S12] D. J. Passos, G. B. Ventura, J. M. V. P. Lopes, J. M. B. L. d. Santos, and N. M. R. Peres, Nonlinear optical responses of crystalline systems: Results from a velocity gauge analysis, Phys. Rev. B **97**, 235446 (2018), arXiv:1712.04924 [cond-mat.other].
[S13] S. M. João and J. M. Viana Parente Lopes, Basis-independent spectral methods for non-linear optical response in arbitrary tight-binding models, Journal of Physics Condensed Matter **32**, 125901 (2020), arXiv:1810.03732 [cond-mat.other].
[S14] H. Rostami, M. I. Katsnelson, G. Vignale, and M. Polini, Gauge invariance and Ward identities in nonlinear response theory, Annals of Physics **431**, 168523 (2021), arXiv:2102.04425 [cond-mat.mes-hall].
[S15] B. A. Bernevig and T. L. Hughes, *Topological Insulators and Topological Superconductors* (Princeton University Press, 2013).
[S16] C. Aversa and J. E. Sipe, Nonlinear optical susceptibilities of semiconductors: Results with a length-gauge analysis, Phys. Rev. B **52**, 14636 (1995).
